# Neural Features of Processing the Enforcement Phrases Used during Occupational Health and Safety Inspections: An ERP Study

**DOI:** 10.3389/fnins.2016.00469

**Published:** 2016-10-19

**Authors:** Qingguo Ma, Liping Shi, Linfeng Hu, Qiang Liu, Zheng Yang, Qiuzhen Wang

**Affiliations:** ^1^Institute of Neural Management Sciences, Zhejiang University of Technology Hangzhou, China; ^2^Neural Industrial Engineering Laboratory, Harbin Engineering University Harbin, China; ^3^Neuromanagement Laboratory, Zhejiang University Hangzhou, China; ^4^Department of Business and Management, School of Economics and Management, Harbin Engineering University Harbin, China; ^5^Department of Data Science and Engineering Management, School of Management, Zhejiang University Hangzhou, China; ^6^Department of Business Administration, School of Economics, Liaoning University of Technology Jinzhou, China; ^7^Department of Management Science and Engineering, School of Economics and Management, Harbin Engineering University Harbin, China

**Keywords:** occupational health and safety, P300, attention, event-related potentials, neuromanagement

## Abstract

The appropriate enforcement phrases used during occupational health and safety (OHS) inspection activities is a crucial factor to guarantee the compliance with OHS regulations in enterprises. However, few researchers have empirically investigated the issue of how enforcement phrases are processed. The present study explored the neural features of processing two types of enforcement phrases (severe-and-deterrent vs. mild-and-polite phrases) used during OHS inspections by applying event-related potentials (ERP) method. Electroencephalogram data were recorded while the participants distinguished between severe-and-deterrent phrases and mild-and-polite phrases depicted in written Chinese words. The ERP results showed that severe-and-deterrent phrases elicited significantly augmented P300 amplitude with a central-parietal scalp distribution compared with mild-and-polite phrases, indicating the allocation of more attention resources to and elaborate processing of the severe-and-deterrent phrases. It reveals that humans may consider the severe-and-deterrent phrases as more motivationally significant and elaborately process the severity and deterrence information contained in the enforcement phrases for the adaptive protection. The current study provides an objective and supplementary way to measure the efficiency of different enforcement phrases at neural level, which may help generate appropriate enforcement phrases and improve the performance of OHS inspections.

## Introduction

Nowadays, the occupational health and safety (OHS) issue has attracted more and more attention from the enterprises, governments, as well as researchers because of its close relationship to the vital interests of the workers and great importance to enterprises' operation and performance (Fernández-Muñiz et al., 2009). Among multiple factors influencing the implementation of OHS management system in the enterprises, the OHS inspections from the enforcement agencies play an important role in ensuring the enterprises' compliance with OHS legislation and guaranteeing the workers' OHS in good status (Niskanen et al., 2014; Hale et al., 2015; Kvorning et al., 2015). The enforcement phrases used by inspectors have a direct impact on the workers and managers' responses to enforcement actions, which is a key factor to make the regulations of OHS practicable and encourage the compliance (Fernández-Muñiz et al., 2009; Kvorning et al., 2015). Generally, the enforcement phrases can be broadly categorized into severe-and-deterrent phrases and mild-and-polite phrases according to two enforcement strategies (deterrence and punishment strategy or advice and persuasion strategy; Niskanen et al., 2014; Hale et al., 2015). Until now, few researchers have studied the cognitive processing of the enforcement phrases and the differences in processing between the two types of phrases. It is still not clear whether the severity and deterrence information are conveyed by the enforcement phrases and humans can identify them. The aim of this study is to address these issues at the neural level.

Emotional stimuli always capture more attention and are elaborately processed since they are important for human's survival, preservation and reproduction (Lang et al., 1997; Schupp et al., 2007). Words/phrases, pictures and facial expressions are commonly used as the stimuli to study the emotion processing (Schupp et al., 2004; Olofsson et al., 2008; Citron, 2012). As a powerful communication way, words or phrases are efficient to convey emotion contents and cognitive contents, which are valuable for human's life, for example, alert humans to hazard or risk in a hazardous environment (Qin and Han, 2009; Ma et al., 2010; Citron, 2012; Shang et al., 2015). Some words directly express the emotional state (e.g., happy, sad) while others denote the emotional connotation (e.g., reward, danger; Citron, 2012). Several studies find words/phrases with emotional connotation do not merely modulate the allocation of attention, they can also convey some valuable information that lead to further cognitive processing (Qin and Han, 2009; Ma et al., 2010; Shang et al., 2015). For instance, the hazard information contained in the warning signal words (e.g., danger, urgent; Ma et al., 2010) and the environment risk information embedded in the words/phrases depicting environmental events (e.g., earthquake, air pollution; Qin and Han, 2009) can be identified and further evaluated by humans. In order to explore the cognitive mechanisms underlying the emotional stimuli processing, many studies apply the event-related potentials (ERPs), which provides high temporal resolution and allows a finer examination of the amount of time and resources allocated to stimuli evaluation (Hillyard and Kutas, 1983). One of the ERP component associated with the emotional stimuli processing is the P300, which is a positive ERP component with a peak latency at around 300 ms after the onset of stimuli (Polich, 2007; Olofsson et al., 2008; Citron, 2012). Multiple studies indicate that P300 demonstrates the neural activity associated with cognitive operations and larger amplitude reflects the more attention resources engaged for stimuli processing (Kok, 2001; Polich, 2007; Schupp et al., 2007; Shang et al., 2015). Previous ERP studies about the emotion processing reveal that high-arousal stimuli often elicit enlarged P300 than low-arousal stimuli, indicating more attention resources are allocated to process the high-arousal stimuli (Delplanque et al., 2006; Olofsson et al., 2008; Citron, 2012; Recio et al., 2014; Delaney-Busch et al., 2016). Ma et al. (2010, 2014) categorized the warning signal words and pictures into high-hazard and low-hazard groups according to the arousal strength rating and found high-hazard group evoked larger P300 amplitude in the task of judging the stimuli as high-hazard or low-hazard. Moreover, in an implicit task without emotional categorization, the threatening faces (Schupp et al., 2004) with high-arousal level also elicited augmented P300 compared to the neutral and friendly face expressions that were less arousing. Such effect is also observed in other emotion processing studies using various implicit tasks (e.g., lexical decision task; Schupp et al., 2007; Scott et al., 2009; Citron, 2012; Delaney-Busch et al., 2016). These findings suggest that arousal effect on the attention allocation reflected by enlarged P300 exists in both passive viewing and active response tasks (Olofsson et al., 2008).

The most methodologies used in the OHS empirical studies are the survey and interview, which are based on the self-report data (Fan et al., 2014). To our knowledge, there are few studies using the electrophysiology method, such as ERP, to explore cognitive processes underlying the human's processing of enforcement phrases in OHS area. Based upon the previous studies that suggest words and phrases can convey cognitive contents (e.g., hazard and risk information; Qin and Han, 2009; Ma et al., 2010), we assumed that enforcement phrases applied by inspectors during the OHS inspections may convey severity and deterrence information of different arousal levels and the severe-and-deterrent enforcement phrases were high-arousal stimuli, which contain more obvious severity and deterrence information than mild-and-polite phrases. Thus, more attention resources would tend to be devoted to elaborately process the severe-and-deterrent enforcement phrases, evoking larger P300 than the mild-and-polite phrases. In order to examine this assumption, we conducted an ERP experiment with the task of judging whether the enforcement phrase was severe-and-deterrent or not.

## Materials and methods

### Participants

Sixteen right-handed students (9 females; mean age: 21.50 years, *SD* = 2.03) in Zhejiang University participated in this experiment. All participants had normal or corrected-to-normal vision. None of them reported any history of psychiatric or neurological disorders. They were fully informed of the experiment procedure and provided written consent. They obtained adequate remuneration after the experiment. The study was approved by the Neuromanagement laboratory's ethics committee in Zhejiang University.

### Experimental stimuli

First, we selected 60 enforcement phrases used in OHS inspections from the regulations and documents (e.g., Law enforcement behavior standards of administrative personnel, Civilization Law enforcement terms norms of urban management, and Note of workplace occupational health supervision and inspection provisions) issued by the central government or some local governments. Second, we recruited sixty participants who would not participate in the ERP experiment to assess the perceived arousal strength of severity and deterrence of each enforcement phrase on a five-point Likert scale (1 = the most mild and polite, and 5 = the most severe and deterrent). Last, we chose 30 enforcement phrases out of the initial 60 and categorized them into two groups according to their rating scores. One group comprised 15 severe-and-deterrent phrases (e.g., “you are imposed with a disciplinary warning as a sanction,” “eliminate hazards immediately,” “we will execute compulsory inspection,” etc.) with an average of 3.653 (SD = 0.611), while the other group comprised 15 mild-and-polite phrases (“Hello! Please show your ID,” “please send someone to help with inspection,” “please confirm and sign your name,” and so forth) with an average of 2.660 (SD = 0.518). The alpha reliability coefficients were 0.891 for the severe-and-deterrent group and 0.819 for the mild-and-polite group. The difference in rating scores between the two groups was significant (*t* = 12.08, *p* < 0.001). The number of Chinese characters in each phrase was five to seven and was not significantly different between the two groups [mean number of characters: 6.67 (severe-and-deterrent) vs. 6.53 (mild-and-polite); *t* = 0.642, *p* = 0.526].

### Procedure

During the experiment, participants sat comfortably in an electrically shielded and acoustically isolated room, while their electroencephalogram (EEG) was simultaneously recorded. The enforcement phrases were displayed on a 17″ CRT screen 1 m away from them. The stimuli were presented by the E-Prime 2.0 Software Package (Psychology Software Tools, Pittsburgh, USA) and they subtended ~6.5° × 6.2° of visual angle. Each participant completed 90 trials in the experiment. In each trial, a black fixation cross was presented for 500 ms at the center of the screen followed by a random interval between 500 and 700 ms. Then, an enforcement phrase was presented for 2000 ms. Once the phrases appeared, participants were required to press the key button 1 or 3 to indicate the group (the severe-and-deterrent or the mild-and-polite) of the phrases as soon as possible. The key button corresponding to each group was counterbalanced across participants. Each phrase was in Chinese Song typeface with black color against a gray background and was presented for three times. Finally, a gray screen appeared for 500 ms before the next trial. The sequence of the 90 trials was random. Before the main ERP experiment, the participants practiced for ten trials. The whole experiment would not last for more than 6 min.

### EEG recording

EEG was continuously recorded with a Neuroscan Synamp2 Amplifier (Scan 4.3.1, Neurosoft Labs, Inc. Virginia, USA) with 64 Ag/AgCl electrodes positioned according to the International 10–20 System (band pass: 0.05–100 Hz; sampling rate: 500 Hz). All electrodes were first referenced to the left mastoid and later digitally re-referenced to the linked mastoids. Vertical and horizontal electrooculograms (EOGs) were monitored with two pairs of electrodes. One pair was placed above and beneath the left eye in parallel with the pupil, and the other at the outer canthus of each eye. All electrode impedance was maintained below 5 kΩ.

### Data analysis

Offline analysis of the recorded EEG data included the following procedures by using Scan 4.5 software (Compumedics NeuroScan Inc., Herndon, Virginia, USA): vertical ocular artifact correction using the regression approach described by Semlitsch et al. (1986), digitally low-pass (30 Hz, 24 dB/Octave) filtering, segmenting EEG data into epochs of 1000 ms (from 200 ms before to 800 ms after the phrases stimuli onset), baseline correction (data in the pre-stimuli period served as baseline). Epochs with peak-to-peak deflection exceeding ±80 μV were excluded from later ERP averaging. Finally, the data were averaged separately for severe-and-deterrent phrases and mild-and-polite phrases.

**Figure 1:**
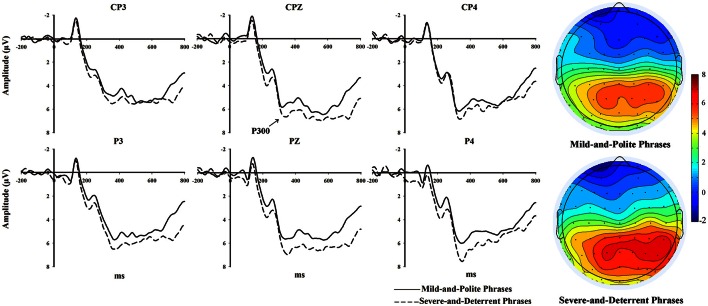
**The grand average waveforms of P300 and topographic maps in 320–400 ms time window**.

Similar to previous related studies (Kok, 2001; Olofsson et al., 2008), we observed the P300 component mainly distributed over the central-parietal regions (see the topographic maps in Figure 1). On the basis of visual inspection, P300 was measured as the mean amplitude of the time window from 320 to 400 ms, and we selected 6 electrodes of CP3, CPz, CP4, P3, Pz, and P4 for statistical analysis. Within-participant repeated-measures analyses of variance (ANOVAs) were performed to examine the effects of phrase type (severe-and-deterrent vs. mild-and-polite) and electrode on the P300 component. The Greenhouse-Geisser correction was applied for the violation of the sphericity assumption in ANOVA [uncorrected degrees of freedom are reported with corrected *p*-values and epsilon values (ε)], and multiple comparisons were corrected with the Bonferroni method when appropriate.

## Results

### Behavioral results

The paired *t*-test showed that the reaction time was not significant between severe-and-deterrent enforcement phrases and the mild-and-polite enforcement phrase (*t* = −1.929, *p* = 0.073), although the reaction time to distinguish severe-and-deterrent phrases was longer (Mean = 960.315 ms, SD = 113.921 for severe-and-deterrent; Mean = 903.058 ms, SD = 166.158 for mild-and-polite).

### ERP results

The grand average waveforms of P300 are shown in Figure 1. The dashed line represents the severe-and-deterrent enforcement phrases and the solid line represents the mild-and-polite enforcement phrases. The 2 (Phrase type: severe-and-deterrent vs. mild-and-polite) × 6 (Electrodes: CP3, CPz, CP4, P3, Pz, P4) repeated-measures ANOVAs showed that the main effect of phrase type was significant [*F*_(1, 15)_ = 8.088, *p* = 0.012]. The enforcement phrases in the severe-and-deterrent group elicited larger P300 than the phrases in the mild-and-polite group did (mean = 7.6270, SD = 4.4243 for severe-and-deterrent phrase; mean = 6.6941, SD = 3.8164 for mild-and-polite phrase). The electrode effect was not significant (*p* > 0.1).

## Discussion

The present study investigated the neural activities underlying the processing of two types of enforcement phrases (severe-and-deterrent vs. mild-and-polite) used during the OHS inspections by means of an explicit evaluation task (judging the phrase as severe-and-deterrent vs. mild-and-polite). Behavioral results showed that the reaction times of distinguishing these two types of enforcement phrases were not significantly different. As for ERP results, both the severe-and-deterrent enforcement phrases and the mild-and-polite phrases evoked P300 waveforms with a central-parietal scalp distribution. The severe-and-deterrent phrases elicited a significantly augmented P300 than the mild-and-polite phrases did. It indicated that the participants devoted more attention resources to elaborately process the severe-and-deterrent phrases.

P300 component is regarded as a neural index of intensity of stimuli evaluation and measurement of attention resource allocated to the task or stimuli (Kok, 2001; Polich, 2007; Schupp et al., 2007). A classic finding is that motivationally significant stimuli (e.g., emotional value and task-relevance) receive preferential attention to be processed and thus elicit enlarged P300 (Kok, 2001; Schupp et al., 2007). In most ERP studies regarding the emotion processing, larger P300 amplitude is evoked by high-arousal stimuli than low-arousal ones (Delplanque et al., 2006; Olofsson et al., 2008; Citron, 2012; Recio et al., 2014; Delaney-Busch et al., 2016). This preferential attention and elaborate processing may result from the high intrinsic motivational properties of the high-arousal stimuli (e.g., high hazard, risk, and threat), which enhance the encoding processing of the stimuli (Lang et al., 1993; Azizian and Polich, 2007; Olofsson et al., 2008). High-arousal stimuli are seen as motivationally significant insofar as they are relevant to human's survival, preservation, and reproduction (Lang et al., 1997; Schupp et al., 2007; Kousta et al., 2009). For example, risky environment events such as floods and earthquakes would lead to catastrophic consequences, which are relevant to the survival of a lot of people. Hence, the words or phrases depicting such risky events induce more brain activation (a larger central-parietal late positive potential) for processing than that depicting safe events (Qin and Han, 2009). Other high-arousal stimuli including warning words (Ma et al., 2010; Shang et al., 2015), hazardous pictures (Ma et al., 2014), and threatening faces (Schupp et al., 2004) are relevant to human's safety and thus elicit larger P300. Qin and Han (2009) suggested that human can extrapolate the severe and dreadful consequence associated with the risky environmental event depicted by words/phrases and retrieve related emotional experience. Ma et al. (2010, 2014) found humans could evaluate the hazard level of hazardous stimuli and had a stronger emotion reaction when confronted with high-hazard stimuli. In present study, the severe-and-deterrent phrases used to enforce the inspections of OHS have higher arousal level of severity and deterrence than mild-and-polite phrases. These obvious severity and deterrence information conveyed by the severe-and-deterrent enforcement phrases may lead to the dread about severe and punitive consequences inferred from the phrases if violating the regulations and are important for the rapid modifications of behavior for preservation or protection (Lang et al., 1997; Kousta et al., 2009). This motivationally relevant information can be identified by the humans and occupy more attention resources to be processed elaborately. Therefore, severe-and-deterrent enforcement phrases are regarded as motivationally significant stimuli and are more likely to provoke the humans' motivation system in the brain compared with mild-and-polite phrases, represented by enlarged P300. In accordance with previous ERP studies related to the emotional stimuli processing (Keil et al., 2002; Schupp et al., 2007; Olofsson et al., 2008), we also found a central-parietal scalp distributed P300 for the processing of both severe-and-deterrent and mild-and-polite phrases at later stage.

Before the current study, there were only survey and interview studies with respect to OHS and few explored the cognitive mechanism underlying the processing of different enforcement phrases used during OHS inspections. This study, for the first time, explores the cognitive processes involved in enforcement phrases processing by using ERP measure. We find the P300 is sensitive to the severity and deterrence level and suggest this neural indicator may serve as an objective and supplementary way to measure the efficiency of enforcement phrases and guide the generation of appropriate enforcement phrases, which should reach enough severity and deterrence level.

In sum, the current study indicates the different neurocognitive processes involved in the processing of severe-and-deterrent phrases and mild-and-polite phrases used by inspectors of OHS. Severe-and-deterrent phrases convey higher level of severity and deterrence and are regarded as more motivationally significant. Therefore, such phrases attract more attention and have the advantage to be processed elaborately compared with the mild-and-polite phrases, reflected by augmented P300 amplitude. It reveals that humans indeed process the severity and deterrence information contained in the enforcement phrases. Besides, our study first shows the possibility of application of neuroscience tools (electrophysiological measurement) in the safety science to measure the efficiency of enforcement phrases and improve the quality of inspections and OHS performance.

## Author contributions

QM, LS, and QW conceived and designed the experiments. QL, LH, and ZY performed the experiment. LH and QL analyzed the data. QM, LS, and LH wrote and refined the article.

## Funding

This work was supported by grant No. 71371167 and No. 71271063 from the National Natural Science Foundation of China and No. AWS14J011 from the National Project.

### Conflict of interest statement

The authors declare that the research was conducted in the absence of any commercial or financial relationships that could be construed as a potential conflict of interest.
