# Corrigendum: Neural Features of Processing the Enforcement Phrases Used during Occupational Health and Safety Inspections: An ERP Study

**DOI:** 10.3389/fnins.2019.00166

**Published:** 2019-03-05

**Authors:** Qingguo Ma, Liping Shi, Linfeng Hu, Qiang Liu, Zheng Yang, Qiuzhen Wang

**Affiliations:** ^1^Institute of Neural Management Sciences, Zhejiang University of Technology, Hangzhou, China; ^2^Neural Industrial Engineering Laboratory, Harbin Engineering University, Harbin, China; ^3^Neuromanagement Laboratory, Zhejiang University, Hangzhou, China; ^4^Department of Business and Management, School of Economics and Management, Harbin Engineering University, Harbin, China; ^5^Department of Data Science and Engineering Management, School of Management, Zhejiang University, Hangzhou, China; ^6^Department of Business Administration, School of Economics, Liaoning University of Technology, Jinzhou, China; ^7^Department of Management Science and Engineering, School of Economics and Management, Harbin Engineering University, Harbin, China

**Keywords:** occupational health and safety, P300, attention, event-related potentials, neuromanagement

There is an error in the Funding statement. The correct number for the funder “National Project” is “AWS14J011.”

The authors apologize for this error and state that this does not change the scientific conclusions of the article in any way. The original article has been updated.

